# PABPC1——mRNA stability, protein translation and tumorigenesis

**DOI:** 10.3389/fonc.2022.1025291

**Published:** 2022-12-01

**Authors:** Ya Qi, Min Wang, Qi Jiang

**Affiliations:** ^1^ Department of Gynecology and Obstetrics, Shengjing Hospital Affiliated of China Medical University, Shenyang, Liaoning, China; ^2^ Second Department of Clinical Medicine, China Medical University, Shenyang, Liaoning, China

**Keywords:** cytoplasmic poly-A binding protein (PABPC1), cancer development, tumor progression, mRNA translation, mRNA stability, oncogene

## Abstract

Mammalian poly A-binding proteins (PABPs) are highly conserved multifunctional RNA-binding proteins primarily involved in the regulation of mRNA translation and stability, of which PABPC1 is considered a central regulator of cytoplasmic mRNA homing and is involved in a wide range of physiological and pathological processes by regulating almost every aspect of RNA metabolism. Alterations in its expression and function disrupt intra-tissue homeostasis and contribute to the development of various tumors. There is increasing evidence that PABPC1 is aberrantly expressed in a variety of tumor tissues and cancers such as lung, gastric, breast, liver, and esophageal cancers, and PABPC1 might be used as a potential biomarker for tumor diagnosis, treatment, and clinical application in the future. In this paper, we review the abnormal expression, functional role, and molecular mechanism of PABPC1 in tumorigenesis and provide directions for further understanding the regulatory role of PABPC1 in tumor cells.

## Background

**Figure 1:**
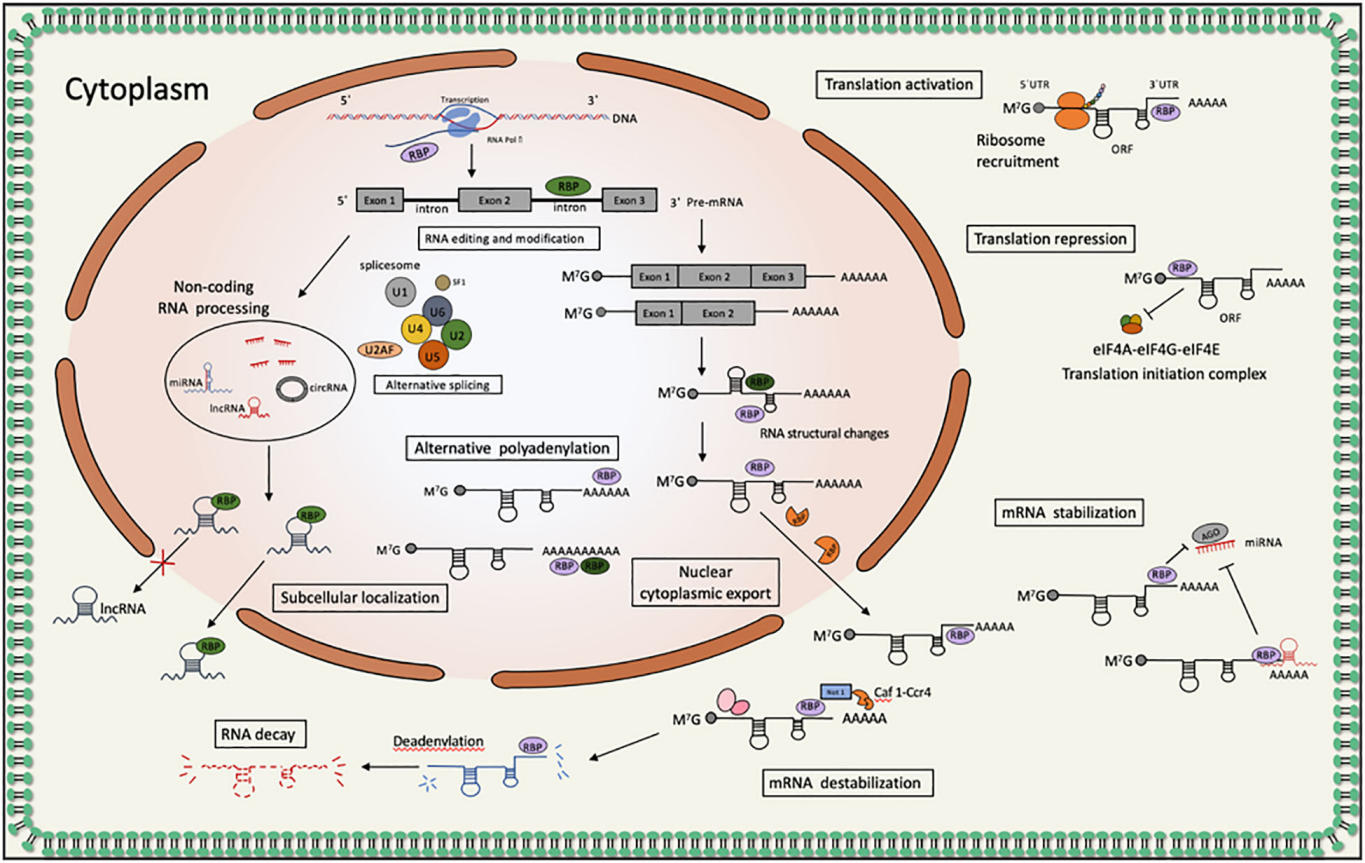
PABPs proteins act as RNA binding proteins involved in post-transcriptional regulation of gene expression in tumors: bind the poly(A) tail of mRNA, including those of their own transcripts, and regulate processes of mRNA metabolism such as pre-mRNA splicing, alternative polyadenylation and mRNA stability; participate in microRNA-induced target gene inactivation and nonsense-mediated mRNA decay; regulate translation initiation and involved in translationally coupled mRNA turnover.

There are about 1914 human RNA binding proteins (RBPs) identified in studies to date, accounting for 7.5% of protein-coding genes (1). RBPs are highly species conserved and play a key role in maintaining homeostasis of gene expression (2). RBPs play a key role in regulating various RNA processes through both temporal and spatial regulation of expression in membranes or phase-separated subcellular compartments of dynamic shuttling, interactions with specific protein partners and RNA targets, and control of all metabolic processes of RNA. This includes splicing, cleavage and polyadenylation as well as translocation, translation and degradation of coding RNAs, non-coding RNAs and microRNAs (3–7). Recent studies have shown that RBPs not only play important roles in normal cells, but also become major players in cancer development and proliferation (8, 9). (**Figure 1**) RBPs were initially classified according to their RNA-binding structural domains that bind various types of RNAs, while they were purified and classified according to the RNA sequences they interact with, with one class of factors including proteins that recognize polyadenylate tails added to the 3’ ends of most mRNAs (10). Poly(A)-binding proteins (PABPs) represent one of the major classes of regulatory proteins and are found only in eukaryotes (11). PABP1 (also called PABPC1), the prototypical PABP, is highly conserved RNA-binding protein in eukaryotes, and although yeast has only one PABPC (Pab1p), most animals contain multiple paralogs with spatially and temporally distinct expression patterns. Vertebrates express PABPC1, PABPC3, PABPC4, PABPC4L, the X chromosome-encoded protein PABPC5 and PABPC1-like (PABPC1L, also known as embryonic PABP (ePAB) in the cytosol and PABPN1 and PABPN1-like (PABPN1L, also known as embryonic PABP2 (ePABP2) in the nucleus. (**Table 1**) PABPC1 and PABPC4 are nucleocytoplasmic shuttling proteins, and RNA is the main factor determining their nucleoplasmic localization. In contrast, nuclear PABPs are structurally and functionally distinct from cytoplasmic PABPs, which are involved in stimulating mRNA maturation and export (10, 12–14).

**Table 1:** The expression and known function of PABPs.

Gene name	Expression	Known functions
PABPC1	Ubiquitous	mRNA Translation, mRNA Decay, miRNA-mediated Repression, NMD, L1 Retrotransposition, mRNA Localization, Local Translation
PABPC3	Testis specific	Spermatid mRNA Translation
PABPC4	T Cells & Other Tissues	Erythroid Differentiation, Upregulated upon T Cell Activation, PABPC1 Compensation
PABPC5	Fetal Brain & Other Adult Tissues	PABPC1 Compensation
PABPC1L(ePAB)	Ovaries, Testes & Other Adult Tissues	Oocyte mRNA Translation
PABPC4L	Brain	PABPC1 Compensation
PABPN1	Ubiquitous	Post-Transcriptional Processing of RNA, Polyadenylation, Poly (A) Tail Length,Poly(A) RNA Export
PABPN1L(ePAB2)	oocytes, early development, and in adult ovarian tissues & Other Tissues	oocyte and preimplantation embryo mRNA degradation and Translation

Protein-coding genes are transcribed by RNA polymerase II production co-transcriptionally mature mRNA precursors. Co-transcriptional maturation of precursor mRNAs is achieved by recruitment of protein complexes through the carboxy-terminal structural domain (CTD) of RNA polymerase II, including the addition of a 5’ cap to the nascent transcript, removal of introns by the spliceosome, and addition of a 3 ‘ poly(A)tail (15). The post-transcriptional addition of 3’ poly(A)tails to eukaryotic mRNAs is important in order to their nuclear export, translation and stability. Poly(A)-binding proteins (PABPs) have the ability to bind poly(A) tails, interact with specific sequences in mRNAs, and have general and specific roles for different mRNA metabolism. PABPs are involved in mRNA in almost all metabolic pathways: polyadenylation/deadenylation, mRNA export, mRNA surveillance, translation, mRNA degradation, microRNA-related regulation and expression regulation during development, especially controlling mRNA-specific translation and stability, accompanying mRNA from initial intranuclear production to final destruction (13, 16). The translation mechanism of most eukaryotic mRNAs is cap-dependent translation, i.e. translation control is dependent on post-transcriptional modifications. Cap-dependent translation initiation involves the binding of the eIF4F complex, consisting of the cap-binding protein eIF4E, the scaffolding protein eIF4G and the the RNA helicases enzyme eIF4A, to the 7-methylguanosine cap located at the 5’ end of the mRNA. MRNA translation also regulates gene expression, which explains the difference between protein abundance and its corresponding mRNA (17–22). In fact, in tumors, changes in the translation of existing mRNAs are more extensive than those occurring in transcription downstream of aberrant signaling pathways (15).

Cytoplasmic poly A binding protein (PABPC1) is significantly highly expressed in a variety of tumors, especially in ovarian, breast, gastric and hepatocellular carcinomas (23–29). PABPC1 regulates the proliferation and transformation of gastric cancer cells *in vitro* and *in vivo* (25), and is also directly involved in breast carcinogenesis by affecting chemoresistance (24). PABPC1, an oncogene in hepatocellular carcinoma, induces cell proliferation by promoting tumor cells into S phase and enhancing anchorage-independent growth (28). LncRNA SNHG14 upregulates PABPC1 through H3K27 acetylation and regulates PTEN signaling in hepatocarcinogenesis to promote tumor cell proliferation and angiogenesis (29). PABPC1 is involved in a variety of signaling pathways involved in tumorigenesis and progression, including Nfr2 signaling, Hippo signaling and PTEN signaling (24, 29, 30), and PABPC1 may be a potential target for tumor therapy.

## Cloning, characterization, and tissue distribution of PABPC1

Although there are numerous PABPs in mammals, almost all studies have focused on the prototype PABPC1. The PABPC1 gene, originally isolated and cloned in human melanoma cells by Grange et al. in 1978, is conserved throughout eukaryotes and is located on human chromosome 8 (31, 32). PABPC1 consists of four RNA binding domains (RRM1-4), a linker region and a C-terminal MLLE domain. RRM1-2 binds poly(A) with high specificity, RRM3-4 binds poly(A) with slightly lower affinity and can bind adenine/uridine rich RNA (33, 34). In addition to RNA, its C-terminal MLLE domain can mediate binding to the peptide motif PAM2, which allows PABPC1 to bind to more proteins such as PAIP1, PAIP2, GW182 and MKRN1 (35–39). Most of these PAM2 motif-containing proteins are involved in mRNA translation and processing, including eukaryotic release factor 3 (eRF3), the deadenylase complex PAN2/PAN3 and TOB1/2 proteins regulating the CCR4-NOT deadenylase complex, and the GW182 protein family. Moreover, the E3 ubiquitin ligase EDD is the only protein other than PABPC1 that contains this MLLE structural domain, suggesting that both appear to have a deeper role in proteostasis (38, 40, 41). Protein expression in different tissues in the Human Protein Atlas dataset (https://www.proteinatlas.org/) showed that PABPC1 was mainly expressed in respiratory system, proximal digestive tract, gastrointestinal tract. Among them, the expression of nasopharynx, esophagus, stomach, urinary bladder, testis,tonsil,bone marrow is the highest, with low tissue specificity. The subcellular localization and expression of PABPC1 were mainly concentrated in the cytoplasm, suggesting that PABPC1 is involved in mRNA stability and translation regulation. PABPC1 can shuttle between the nucleus and cytoplasm (42), while at steady state, it is mainly diffusely distributed in the cytoplasm (13, 42). In addition to localization in the cytoplasm and nucleus, PABPC1 is also present at local translational sites such as neuronal dendrites and is enriched at the leading edge of migrating fibroblasts (43). In contrast, mRNA-bound PABPC1 cannot enter the nucleus because its RRM cannot interact with importinβ (44). PABPC1 is not always uniformly distributed in the cytoplasm but assembles into dynamic non-membrane foci that are incorporated into stress granules during cellular stress conditions such as osmotic shock (45), and these stress granules are sites of mRNA storage and remodeling by stalled translation initiation complexes composed of mRNA, small ribosomal subunits, specific initiation factors, and many mRNA-binding proteins.

## The interactome of PABPC1

All aspects of life require tight regulation of gene expression, which is regulated through a complex interconnected network that includes multiple levels of transcription, mRNA processing, mRNA stability, and mRNA translation. Post-transcriptional control mechanisms are complex and important. The regulation of post-transcriptional gene expression is regulated by both mRNA cis-elements (e.g. 5’- and 3’-untranslated regions) and trans-acting factors (e.g. RBPs) (7). They form RNA-protein complexes through specific interactions to regulate mRNA metabolism and protein translation.

### PABPC1 and mRNA translation

Most eukaryotic mRNAs are recruited to the ribosome by recognition of a 5’m7GpppN cap. One model assumes that mRNAs form a closed loop structure that involves 5’-3’ end proximity induced by four specific interactions, namely cap-eIF4E- eIF4G-PABPC1-poly(A). The 5’ cap interaction factor and the 3 ‘poly(A) binding protein plays an important role in bringing the 5’-3’ ends of mRNAs in close proximity to each other and promoting translation and stability of mRNAs (46–48). This mode of translation is treated as a general model of eukaryotic translation in today’s biology textbooks. However, despite the direct observation of loop assembly in some cells and *in vitro* reconstruction systems, several findings have questioned the generality of the model across different mRNA and biological systems (49–52).

Translation of most mRNAs is regulated at the time of initiation. At translation initiation, eIF4E binds to the 5’ cap while PABPC1 binds to the poly(a) tail, and eIF4G binds the two together to form a complex. Then eIF3 and the 43s ribosomal subunit bound to met-tRNA bind to the complex, initiating translation initiation. eIF3 is an initiation factor that functions by facilitating the linkage of the 48s initiation complex to the 60s subunit. Transmission electron microscopy that several PABPC1 molecules in the PABPC1- poly(a) complex were linearly arranged to form a worm-like structure. Further enhancement of translation was reported with the increasing length of poly(a), which seems to be associated with an increase in the number of PABPC1 molecules in the polyplex bound to poly(a) (53). The tethered structural domain of PABPC1 stimulates translation initiation, suggesting that the poly(a) length dependence of translation is due to the increased availability of PABPC1 bound at the 3’ end of the mRNA. The long poly(a) tails on most vertebrate mRNAs function as enhancers of translation initiation by recruiting multiple PABPC1 to the 3’ ends of mRNAs (54). Borman et al. extracted mammalian cytoplasm and partially depleted ribosomes and associated initiation factors by ultracentrifugation. Replication of the cap-poly(A) synergistic system *in vitro* demonstrated that PABPC1 enhances translation on poly(a) of mRNA in a cap-dependent manner, in addition to finding that binding of PABPC1 to poly(A) increased the affinity of PABPC1 for eIF4G and eIF4E for cap, which in turn increased translation initiation. Consistent with this, mutations that eliminate or alter PABPC1-eIF4G interactions result in reduced translation levels (55, 56). Unfertilized eggs and early embryos of animals lack the ability to produce or destroy mRNA, so for the regulation of protein production, they cannot adjust the number of mRNA molecules used for translation, but rather by changing the length of the poly(A) tail at the end of each mRNA molecule. In animal oocytes and early embryos, the poly(A) length of mRNAs strongly affects translation efficiency, with mRNAs with longer poly(A) tails being more efficiently translated than shorter ones, but this linkage gradually disappears later in development. Artificially giving higher levels of PABPC in frog eggs improves translation of the short tails and even achieves the same translation efficiency as long-tailed mRNAs, suggesting that there is competition for PABPC between the two (53).

The role of PABPC1 in stimulating translation initiation was demonstrated (57, 58); however, the role of PABPC1 in translation is not limited to regulating initiation. Using a reconstructed mammalian *in vitro* translation system, Ivanov et al. found that PABPC1 directly stimulates translation termination (59, 60). PABPC1 stimulates translation termination through the interaction of its C-terminal structural domain with eukaryotic peptide release factor 3 (eRF3). Eukaryotic translation termination requires two release factors: eukaryotic polypeptide release factor (eRF1)1 and eRF3 (61). ERF3 is a GTPase with an intrinsic PAM2 motif that binds to PABPC1, and a protein core (eRF3c) that interacts with eRF1 to ensure that the latter is loaded on the ribosome and induces its conformational rearrangement. The binding action of PABPC1 to eRF3 enhances the loading of the release factor on the ribosome, thereby preventing the interaction of the eRF1-eRF3 complex with the nonsense-mediated decay mechanism and inhibiting the reading of the termination codon. The C-terminal structural domain of PABPC1 interacts with the N-terminal structural domain of eRF3a to enhance the binding of the eRF1-eRF3 complex to the ribosome, while eRF3a itself plays an active role in translation termination (62–67).

In addition, PABP-interacting proteins 1 and 2 (PAIP1 and PAIP2), bind the same structural domains of PABPC1 and regulate its translational activity (68). The results of Ivanov et al. showed that both PAIP1 and PAIP2 prevent translation termination due to premature stop codons by controlling PABPC1 activity (59).PAIP1 contains PAM1 and PAM2 motifs, and a structural domain homologous to the mediate eIF4G structural domain (meIF4G) (69), whereas PAIP2 contains only the PAM1 and PAM2 motifs. PAIP1 binds the RRM1-RRM2 motif of PABPC1 with high affinity, whereas PAIP2 binds the CTC structural domain with low affinity. PAIP1 is able to induce translational activity by binding to eIF3, eIF4A and PABPC1 simultaneously. In contrast, PAIP2 binds PABPC1 competitively with PAIP1 or eIF4G, reducing the binding affinity of PABPC1 to the poly(A) tail, and therefore, PAIP2 inhibits translation *in vivo* or *in vitro (* 70, 71). Although PAIP2a has two PABPC1 interaction motifs, PAM1 and PAM2, at its N-terminal and C-terminal ends, respectively, PAM1 binds mainly to RRM2-RRM3 with hundreds of times the binding affinity of PAM2 and plays a major role mainly in PAIP2a-mediated dissociation of PABPC1 from poly(A). Recent studies have shown that PAIP2a competitively inhibits the binding of RRM to poly(A), first by binding to RRM2 to form a transient ternary complex and then by replacing poly(A) by RRM3.

Notably, Bartel et al. suggested that while PABPC1 plays a critical role in protecting mRNA from premature decay, its contribution to translation in postembryonic mammalian cell lines is minimal (53). Previous studies have identified a role for PABPC1 in promoting translation, either in frog oocytes or early embryos, *in vitro*, or in cell extracts, while in rabbit reticulocyte lysates, PABPC1 had the least effect on translation (13, 57, 72–74). So further experiments are needed to determine whether this difference is due to cell type or to differences between cell cytoplasm and ex vivo extracts. It is also necessary to distinguish whether it is due to PABPC1 promoting translation activation or mRNA stability.

### PABPC1 and mRNA stability

The mRNAs of different genes are degraded at significantly different rates, as short as a few minutes or as long as several days (75). Different conditions or developmental environments also alter the rate of degradation, affecting the dynamic accumulation of mRNA and ultimately the steady-state abundance of mRNA (75, 76). Termini often determine the fate of the RNA molecules, and 3’ tailing(non-templated nucleotide addition at the 3’ end of RNA) is one of the most common types of RNA modifications, with tailing catalyzed by a set of non-templated terminal nucleotidyltransferases (TENTs) (77, 78). In addition to the attachment of poly(A) polymerase-catalyzed poly(A) tails to mRNA in the form of transcriptional coupling (79), post-transcriptional modifications such as guanylation and uridylation are included to control RNA stability and activity (80, 81). Several cis-acting elements present in the 3’ untranslated region of target mRNAs have been shown to be involved in regulating polyadenylation/de-enylation of specific mRNAs (39). These cis-acting sequences are recognized by microRNAs, AU-rich element-binding proteins, polyadenylation factors or other RNA-binding factors that greatly influence mRNA fate and ultimately gene expression (38, 82, 83).

Bartel et al. showed that almost all endogenous mRNAs are degraded primarily by a mechanism of deadenylation linkage, implying that the rate of deadenylation of each mRNA largely determines its half-life, while other contributions such as endonucleolytic cleavage and deadenylation-independent decapping are unexpectedly little (84). Further use of mathematical models to extend the range of known deadenylation rate constants from 60-fold to 1000-fold emphasizes that mRNAs with faster deadenylation rate constants can reach the short tail length associated with mRNA destruction more quickly, while mRNAs with the same 20-nt tail but from different genes can also have widely different decay rate constants. The tails become so short that they cannot synergistically bind PABPC1 and homeostasis is out of balance.

Recent studies have revealed the presence of noncanonical other forms of mRNA tailing, such as U tails and mixed tails (85). Initially uridylation of mRNA was found at the 3’ end of miRNA-mediated cleavage products (86), and U tails have also been detected on human replication-dependent histone mRNAs, which are uridylated and degraded at the end of the S period or after inhibition of DNA replication (87). Lim et al. developed a method called TAIL-seq for deep sequencing of a large portion of the 3’ end sequences of the transcriptome and found that the vast majority of mammalian mRNAs are subject to uridylation (88). Upon deadenylation, mRNAs (with A-tails shorter than ∼25 nt) lose PABPC1 and instead gain a U-tail by the redundant action of TUT4 and TUT7, which triggers decay by serving as a mark that is recognized by downstream decay factors (89). PABPC1 preferentially protects the long poly(A) tail from uridylation, and this specific inhibition may result from length-dependent binding of PABPC1 (90, 91).

The family of La-related proteins (LARPs) is characterized by sharing a ‘La-module’ (92): it contains a La motif (LaM) followed by an RNA recognition motif (RRM), and some LARPs have unique activities in specific aspects of RNA metabolic processes. Among them, LARP3 and LARP7 is nuclear and protects a subset of RNAs with 3′ oligo(U) (93–95) while LARP 1, 4 and 6 are highly divergent and reside in the cytoplasm (96). LARP1 and LARP4 were recently found to contain a PAM2 motif that binds directly to poly(A) and PABPC1 with variable affinity for the MLLE structural domain of PABPC1. LARP4 is an mRNA poly(A) stabilizer that promotes mRNA translation and inhibits deadenylation. LARP1 is a translation blocker that inhibits translation of mRNAs containing 5 ‘TOP (terminal oligopyrimidine) motifs in mRNA translation and exhibits similar poly(A) length and mRNA stability protection to LARP4 (97, 98). Mattijssen et al. confirmed the interaction of LARP1 with PABPC1 independent of RNA by endogenous pull-down in the presence of RNAase I and further verified the binding to PABPC1 in bacterially expressed LARP1. With the addition of Dey680-labeled A20 RNA, the amount of PABPC1 coprecipitated in the His-LARP1 pull-down increased, indicating that the LARP1 interaction between PABPC1 may be enhanced by binding to RNA (99).

It is now generally accepted that RNA-binding proteins negatively regulate gene expression by accelerating the deadenylation of target mRNAs. The most widely studied example is the RNA-binding protein TTP, which binds directly to AREs and recruits CCR4-NOT complexes to accelerate the deadenylation and decay of target mRNAs (100, 101). In marked contrast, positive regulation through polyadenylation has been most extensively studied in the context of early development (102).Spinocerebellar ataxia type 2 (SCA2) gene product, Ataxin-2, a member of the RNA-binding protein Like-Sm (LSm) family, is a cytoplasmic protein that binds and stabilizes many mRNA sequences, which is involved in many aspects of RNA metabolism (103), and it harbors an LSm domain and LSm-associated domain (LSmAD) within the N-terminal half. Yokoshi successfully identified Ataxin-2 as a member of the group of RBPs that target AREs and related elements within the 3′ UTRs of mRNAs to control RNA stability and protein expression (104). Although interaction with PABPC1 might increase the efficiency of Ataxin-2 binding to RNAs, it is dispensable for recognition of the distinct elements by Ataxin-2. In accordance, they found that Ataxin-2 binds preferentially to sites that are close to the polyadenylation site. These results suggest that interaction with PABPC1 stabilizes the binding of Ataxin-2 to RNAs, and more importantly, PABPC1 probably targets A-rich elements within the 3′ UTR, including the polyadenylation signal, in addition to the poly(A) tail. Inagaki et al. showed that Ataxin-2 indirectly recognizes the poly(A) tail through PAM2-mediated contact with PABPC1, which is assumed to increase the binding specificity of Ataxin-2 to the cis-element close to the polyadenylation site of the mRNA and enables the recruitment of a noncanonical poly(A) polymerase (PAPD4) to the polyadenylation site (39).

### PABPC1 and nonsense-mediated decay of PTC-containing mRNAs

The premature termination codon (PTC) is found in approximately 30% of disease-associated mutations, and because it promotes aberrant as well as premature translation termination, mRNA surveillance mechanisms recognize, respond to, and ultimately produce nonsense-mediated mRNA decay (NMD). Nonsense-mediated mRNA decay detects and eliminates erroneous mRNA transcripts with premature termination codons, representing a translation-dependent post-transcriptional mRNA quality control process that prevents the synthesis of truncated, potentially harmful proteins (105–109). Because one-third of all known disease-causing mutations are predicted to produce PTC, NMD is an important regulator of human inherited disease phenotypes.

In mammalian cells PTCs are recognized and NMD is activated if the PTC is located >50 nucleotides upstream of an exon-junction complex (EJC) (110, 111), a protein complex deposited on the mRNA during splicing (112). During splicing in the nucleus, exon-exon junctions are labeled by EJC and these complexes act as NMD activation signals during translation in the cytoplasm. In addition to the EJC-dependent NMD described above, another EJC-independent NMD pathway targets mRNAs with long 3’UTRs (113–116). Three upshift code proteins, UPF1, UPF2, and UPF3, are at the core of the NMD pathway. UPF1 plays a key role in the initiation of NMD and is an ATP-dependent RNA unwinding enzyme, and UPF1, UPF2, and UPF3 interact so that these three proteins can be separated as a complex. After recognition of PTC, a series of events initiate mRNA degradation by recruiting heterodimers SMG5/SMG7 and SMG6 (117, 118).

During eukaryotic translation termination, PABPC1 interacts with ribosome-bound eRF3a to stimulate polypeptide release and subsequent ribosome recycling; however, when PABPC1 interaction with eRF3a is reduced by an unusually long 3’UTR, UPF1 binds to eRF3a and activates NMD. thus, PABPC1 binding at the termination codon binding in the vicinity can inhibit NMD by mimicking the presence of the poly(A) tail (119). The physical distance between the premature termination codon and PABPC1 is a key determinant of PTC recognition, and by increasing the distance between poly(a)-bound PABPC1 and PTC by lengthening the 3’UTR, the normal termination codon can trigger nonsense-mediated mRNA decay (114). Conversely, NMD can inhibit EJC-enhanced NMD by folding the poly(a) tail near the PTC or tethering it to the PTC (120, 121).

Two independent studies have shown that the C-terminal structural domain of PABPC1, which mediates the interaction with eRF3a, is nonessential for the inhibition of NMD-targeted reporter molecules (120, 121). To elucidate which structural domains and interaction regions of PABPC1 are responsible for the NMD repressive effect, Fatscher et al. designed and generated six mutants, including an RRM-containing mutant lacking the C-terminal MLLE structural domain and an MLLE structural domain mutant, and ligated the mutants to the TPI-4MS2-SMG5 reporter gene construct to increase the mRNA abundance of the reporter gene increased to the same extent as that of PABPC1. The results indicate that the MLLE motif of PABPC1 interacting with eRF3a, as well as other interactions involving the MLLE motif of PABPC1, are not strictly necessary for pegging PABPC1 to suppress NMD. In contrast, the introduction of two point mutations into RRM2 of PABPC1 that eliminate binding to eIF4G (PABPC1M161A/D165K) no longer inhibits NMD, and it is the binding of eIF4G but not eRF3a that contributes to NMD inhibition by pegged PABPC1, suggesting the eIF4G-mediated mRNA recycling and ribosome recycling as NMD regulators importance (119)

### Involved in miRNA-induced target gene inactivation

MicroRNAs (miRNAs) are a new class of non-protein-coding endogenous small RNAs that are important regulatory molecules. It regulates gene expression post-transcriptionally through translational repression, mRNA splicing and mRNA decay initiated by rapidly dying genes. miRNAs silence the expression of most eukaryotic transcriptomes and regulate a wide range of biological processes, including cell growth, division and differentiation, as well as metabolism and development (122–124). At the molecular level, miRNA bases are incompletely paired with the 3’ untranslated region of the target mRNA and bind to the Argonute (Ago) protein in its induced silencing complex (miRISC) to inhibit translation or trigger degradation of the mRNA.

TNRC6A, TNRC6B and TNRC6C are members of the GW182 protein family and components of the miRNA-induced silencing complex (125, 126). the TNRC6-PABPC1 interaction is required for effective miRNA-mediated silencing (127). the carboxy-terminal structural domain of the GW182 protein, called the silencing structural domain, includes intermediate and terminal sub-structural domains (128). The intermediate structural domain can be further subdivided into M1 and M2 regions flanked by poly(A)-binding PAM2. PABPC1 assembles the miRISC complex by recruiting GW182 and bringing the death enzyme complex in close proximity to the mRNA. In contrast, TNRC6 promotes the separation of PABPC1 from mRNA thereby exposing its tail end to promote deadenylation, reducing translation efficiency and causing mRNA decapitation and 5’-3’ exonuclease nucleic acid degradation in somatic cells (129).

### PABPC1 and virus replication

Viruses do not have a specific apparatus to encode their own protein synthesis, but rather compete with the host to translate their own viral mRNA, where the most important mechanism of interference is to disrupt translation initiation on polyadenylate mRNA. Active modification of PABPC1 allows viruses to block translation initiation in the host. Depending on the structure of viral mRNA, different RNA and DNA viruses have different mechanisms to manipulate PABPC1 (130–134).

Lentiviruses (HIV-1 and HIV-2), microRNA viruses and cuplaviruses infect cells and cleave PABPC1 into fragments at several predetermined positions between its RNA binding domain and C-terminal structural domain, limiting the translation of host mRNA. In contrast, rotavirus NSP3 displaces and expels PABPC1 in interaction with eIF4G to mediate translation termination (135). It was shown that members of the herpesviridae, eutheroviridae and bunyaviridae relocate PABPC1 to the nucleus after infection (136). In contrast, specific viral proteins for PABPC1 relocalization were also identified in Kaposi’s sarcoma-associated herpesviruses and rotaviruses (45, 137–139).

### PABPC1 and immunoglobulin secretion

There is growing evidence that selective processing of mRNAs, including selective splicing, 3’ selective polyadenylation and mRNA stability/translational regulation, is a major mechanism that promotes protein diversification (140–143). Selective splicing and polyadenylation in eukaryotic cells generate multiple mRNA types with different 3’UTR lengths from the same gene (144, 145). PABPN1 has been identified as a key molecule regulating selective splicing and polyadenylation, and deep sequencing after siRNA knockdown has shown that PABPC1 enhances selective poly(A) at the distal end of pre-mRNA. A site use (146).

Plasma cells are terminally differentiated B cells that produce large amounts of secretory immunoglobulins. And the first identified example of selective polyadenylation is that the 3’ end of immunoglobulin heavy chain (IgH) mRNA is processed in a cell type-specific manner during B-cell differentiation (147, 148). Switching between mIg and sIg is a tightly controlled process regulated by mRNA selective splicing and selectable polyadenylation. Peng et al. reported that hnRNPLL specifically binds to PABPC1 in T cells and plasma cells, and that PABPC1 facilitates the binding of hnRNPLL to immunoglobulin mRNA and regulates the switch from mIgH to sIgH in plasma cells (149). Further protein truncation and mutation experiments demonstrated that RRM1 is the key structural domain mediating the interaction of PABPC1 with hnRNPLL.

## PABPC1 in cancer development

Human and mouse heart cells proliferate immediately after birth, during which PABPC1 expression is enhanced (13, 54). After they grow, cell proliferation is inhibited and PABPC1 expression is reduced. Dengue viruses use PABPC1 in their host cells to transcribe viral mRNA, which leads to viral proliferation. PABPC1 similarly accumulates in cytomegalovirus-infected cells.

**Figure 2:**
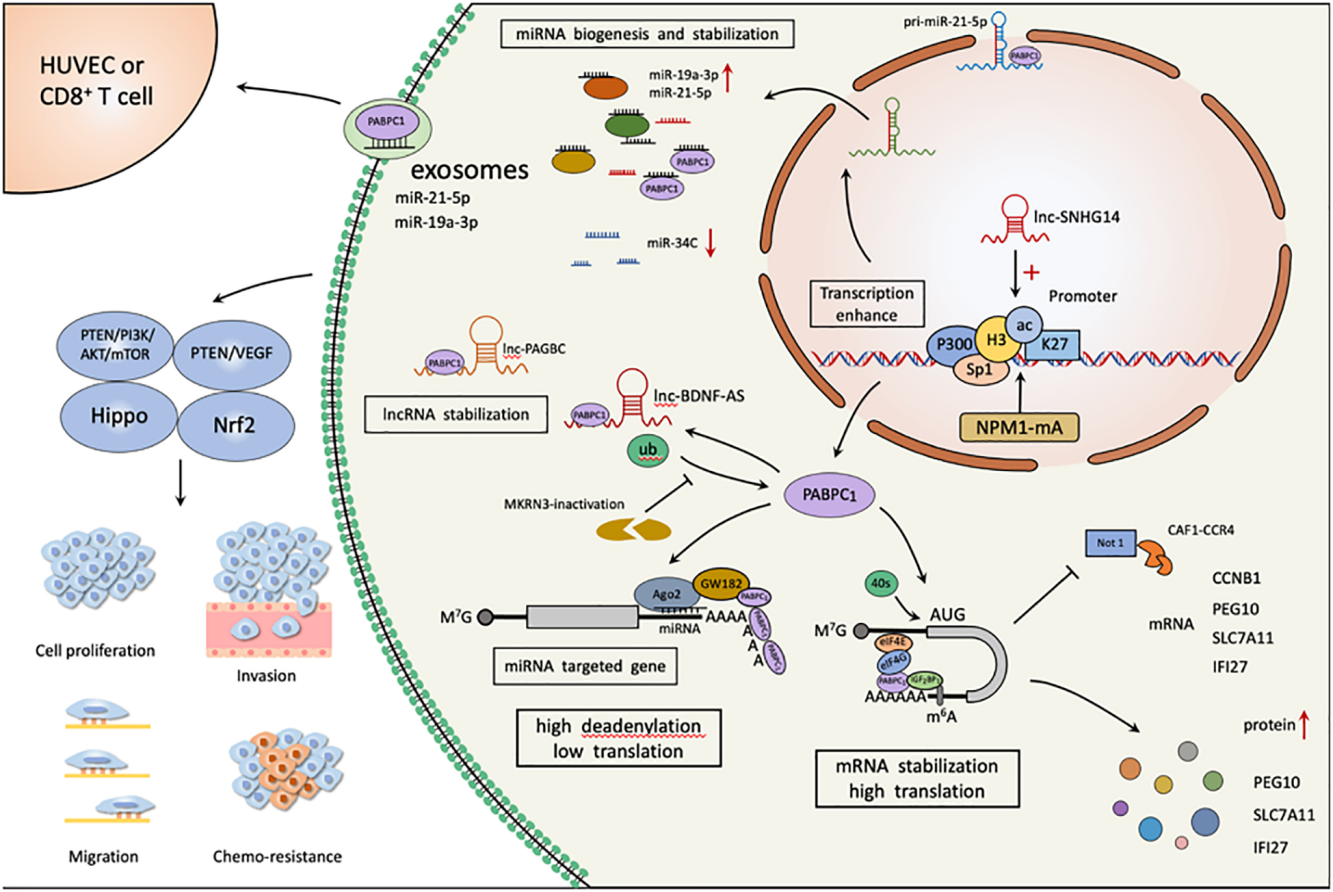
PABPC1 is involved in various aspects of tumor malignant biological behavior as an RNA binding protein. NPM1mA, SNHG14, Sp1 and p300 significantly induce upregulation of PABPC1 expression by increasing acetylation of PABPC1 promoter H3K27 and H2K37; Inactivated MKRN3 reduces PABPC1 ubiquitination, promotes its binding to 3’ poly(A) tails of mRNAs, and thereby accelerates global protein synthesis and promotes cancer proliferation and progression. PABPC1 regulates the generation and stability of tumor-associated RNAs, and the intracellular tumorigenic miR-19a-3p, miR-21-5p, lnc-BDNF-AS, and lnc-PAGBC levels are elevated, along with increased translation of CCNB1, PEG10, SLC7A11, and IFI27, and elevated protein levels. PABPC1 enhances miR-19a-3p, miR-21-5p loading in tumor cell-derived exosomes, target vascular endothelial cells to induce angiogenesis, and CD8+ T cells to impair the immune function.

The expression of PABPC1 in 31 tumor specimens was analyzed using the Tumor Genome Atlas (TCGA) and Tissue Genotype Expression (GTEx) datasets in comparison to normal tissues. Elevated levels were found in acute myeloid leukemia (LAML), esophageal cancer (ESCA), bladder uroepithelial carcinoma (BLCA), gastric cancer (STAD), ovarian cancer (OV), rectal adenocarcinoma (READ), testicular cancer (TGCT), and COAD (colon cancer). High PABPC1 expression was also found in diffuse large B-cell lymphoma (DLBC), head and neck squamous cell carcinoma (HNSC), lung squamous carcinoma (LUSC), prostate cancer (PRAD), and uterine sarcoma (UCS), suggesting that the progression of the above tumors requires high PABPC1 expression (**Figure 2**).

### PABPC1 in liver tumor

Hepatocellular liver cancer is the major malignancy of the liver and is the fifth most common and third deadliest cancer in the world (150, 151). Currently, despite the advances in liver transplantation and surgery in the treatment of liver cancer, the high recurrence and metastasis rates also make the prognosis of liver cancer still poor (152). Therefore, it is crucial to search for the underlying mechanisms of hepatocarcinogenesis and identify new therapeutic targets (153).

Zhu et al. screened gene modules highly correlated with HCC prognosis by WGCNA and constructed PPI networks for the genes in the modules (27). 8 genes were screened using MCODE software and survival analysis was performed using Kaplan-Meier mapping database, showing that PABPC1 was significantly associated with liver cancer prognosis. Further studies confirmed that mRNA and protein levels of PABPC1 were significantly upregulated in HCC patients. GSEA analysis also showed that the P53 signaling pathway, WNT signaling pathway and cell cycle were highly positively correlated with PABPC1 expression. the P53 pathway is one of the classical pathways controlling cell cycle progression and Wnt/β-catenin signaling is involved in a variety of processes including embryogenesis, The P53 pathway is one of the classical pathways controlling cell cycle progression and Wnt/β-catenin signaling plays a key role in the regulation of various processes such as embryogenesis, differentiation and tumorigenesis.

MicroRNAs are involved in hepatocellular carcinoma development and metastasis as a tumor-associated regulator. miRNA-induced RNA-induced silencing complex (RISC) promotes mRNA degradation or translation inhibition. Zhang et al. purified RISC-interacting proteins using anti-AGO2 antibody and identified 12 AGO2-binding proteins by mass spectrometry (28). PABPC1 was found to be highly expressed in hepatocellular carcinoma, especially in high-grade hepatocellular carcinoma. PABPC1 acts as an oncogene in hepatocellular carcinoma, accelerating cell proliferation and promoting anchorage-independent growth by promoting cell entry into S and G2/M phases. The specific mechanism is that PABPC1 interacts with AGO2 in the cytoplasm of hepatocellular carcinoma cells, and this interaction increases the recruitment of mRNA to RISC and represses multiple oncogenes. For miRNA-targeted genes, PABPC1 increases the efficiency of miRNA repression, and this efficiency is higher in cancer cells than in normal cells. For miRNA non-target genes, PABPC1 interacted with eIF4G to inhibit the decay of mRNA, making translation higher in cancer cells than in normal cells and thus increasing cellular activity.

Long non-coding RNAs (lncRNAs) play important roles in various biological processes at epigenetic, transcriptional and post-transcriptional levels, and are of increasing interest for their critical role in tumorigenesis and progression (154–158). Small nucleolar RNA host genes (SNHG) long non-coding RNAs are frequently dysregulated in various types of cancers and are involved in tumorigenesis and progression. Studies have shown that SNHG14 upregulates PABPC1 expression in hepatocellular carcinoma cells *via* H3K27 acetylation (29), and PABPC1 silencing Zhaattenuates SNHG14-induced proliferation and angiogenesis in Hep3B cells, while PABPC1 overexpression abrogates the effect of sh-SNHG14 on HepG2 cell proliferation and angiogenesis. sh-SNHG14/PABPC1 effects on cell proliferation and angiogenesis was regulated by inhibition of PTEN signaling, a tumor suppressor involved in cell proliferation or angiogenesis through negative regulation of PI3K/Akt signaling or VEGF expression, respectively.

Hepatoblastoma is the most common type of liver tumor in children and arises from embryonic parenchymal hepatocytes or hepatoblasts (159, 160). It has a better prognosis compared to hepatocellular carcinoma, but the overall prognosis remains poor for patients who cannot be surgically resected or are chemotherapy resistant (161). m6-methyladenosine (m6A) is considered to be the most abundant mRNA modification in eukaryotic cells and occurs at the N6 position of adenosine (162, 163). As a reversible mRNA modification, it is involved in regulating various aspects of RNA metabolism and also in the oncogenic process. Recent studies have shown that m6A-containing mRNAs exhibit accelerated deadenylation mediated by direct recruitment of the CCR4-NOT complex through YTHDF2 (41, 164). YTHDF2 is has been reported to act as a reader of m6A recognizing and binding specific m6A modifications that also include IGF2BP1-3, YTHDF3 and YTHDC2, among others (165). The study showed that in hepatoblastoma, IGF2BP1 similarly acts as readers to recognize and bind m6A modifications in SLC7A11 mRNA, stabilize it and upregulate its expression in an m6A-dependent manner (26). the CCR4-NOT complex mediates the deadenylation of SLC7A11 mRNA, and IGF2BP1 competitively binds PABPC1 to block BTG2/CCR4-NOT complex recruitment, thereby inhibiting the deadenylation of SLC7A11 mRNA and enhancing SLC7A11 mRNA stability and expression. In contrast, SLC7A11 acts as an oncogene that promotes hepatoblastoma proliferation and enhances ferroptosis resistance in tumor cells.

### PABPC1 in esophageal cancer

Esophageal cancer, the sixth most common cause of cancer-related death worldwide (166), is an aggressive malignancy, of which esophageal squamous cell carcinoma is the predominant histologic subtype, accounting for more than 70% of total cases. Esophageal cancer exhibits local infiltration and lymph node metastasis at advanced or even initial diagnosis, thus leading to poor prognosis and lower survival rates.

Zhang et al. used their own cohort and public database TCGA sample analysis to conclude that PABPC1 expression is upregulated in esophageal squamous cancer tissues and its elevated expression is associated with tumor cell differentiation and poor prognosis in patients (167). Pabpc1 promotes esophageal squamous cancer cell proliferation, migration and invasion and inhibits apoptosis. The IFN pathway was identified by RNA-seq as a key mediator of esophageal squamous carcinoma progression exerted by PABPC1. IFN27 is a key regulator in the IFN-α signaling pathway (168–170) and its upregulation was one of the most significant alterations in DEGs upregulated by PABPC1. The investigators further observed a significant decrease in 5-ethynyluridine (EU)-tagged IFI27 mRNA after PABPC1 knockdown, suggesting that PABPC1 regulates IFI27 expression at the post-transcriptional level. Further construction of RNA-binding structural domain RRM1-deficient plasmids demonstrated that PABPC1 interacts with eukaryotic initiation factor protein 4G (eIF4G) to enhance the stability of IFI27 mRNA by extending its half-life.

In eukaryotic cells, RNA exosomes are essential for the degradation and processing of target RNAs, and the RNA exosome core contains barrel-like structures (composed of EXOSC4-9) and cap-like structures (composed of EXOSC1-3). EXOSC2 is an important catalytic part of the RNA exosome complex, and EXOSC2 knockdown severely reduced RNA exosome function (171, 172). The investigators rescued the decrease in IFI27mRNA stability by EXOSC2 knockdown and EXOSC2 knockdown increased PABPC1 binding to IFI27mRNA, similar to how PABPC1 knockdown increased the binding affinity of EXOSC2 to IFI27mRNA, all suggesting that PABPC1 competes with the RNA exosome to prevent degradation of IFI27mRNA (167). Various miRNAs from exosomes are major inducers of angiogenesis by activating signal transduction pathways that trigger the promotion of endothelial cell growth and migration. PABPC1 increases miR-21-5p expression in esophageal squamous cancer cells and encapsulates miR-21-5p in ESCC cell-derived exosomes *via* ACUGAUG sequences to target vascular CXCL10 inhibition in endothelial cells to induce angiogenesis.

### PABPC1 in leukemia

Tumor-derived exosomes can shuttle between tumor cells and immune cells and participate in tumor immune escape. Exosomal miRNAs contribute to the reprogramming of immune target cell functions. Targeted export of miRNAs to exosomes may require specific mechanisms. Recent studies have shown that RBPs are involved in the specific loading of miRNAs into exosomes. For example, hnRNPA2B1 was shown to enhance the export of miRNA exosomes from T cells. The relevant functions of Ago2 and Y-box in miRNA transport were demonstrated in colon cancer cells and HEK293T cells, respectively. The proteomics of two different populations of exosomes isolated from human seminal plasma showed that PABPC1 could promote RNA loading of exosomes.

Acute myeloid leukemia (AML) is a heterogeneous disease that possesses multiple cytogenetic and molecular abnormalities with an extremely poor prognosis (173). Mutations in the nuclear phosphoprotein gene, particularly the type A NPM1 mutation, are among the most common and clinically relevant genetic alterations. It accounts for approximately 30% of all AML cases (174, 175). As with other malignant diseases, leukemia uses a variety of mechanisms to evade killing by immune cells. T cells play a central role in mediating and coordinating the immune response against cancer, and many strategies aim to harness the potential of t cells to recognize and kill cancer cells in a targeted manner (176, 177). The study found that serum from AML patients with NPM1 mutations and leukemia cells in a co-culture system impaired the immune function of CD8+ T cells (142). Mechanistically, leukemic cells secrete miR-19a-3p into the tumor microenvironment *via* small extracellular vesicles (sEVs), which are controlled by the NPM1 mutant protein/CTCF/PABPC1 signaling axis. SEV-related miR-19a-3p is internalized by CD8+ T cells, directly inhibiting solute carrier family 6 member 8 (SLC6A8)-mediated of creatine import and reduces ATP production to enhance immune escape of leukemic cells. To investigate the specific process of miR-19a-3p specific packaging in sEVs, the investigators found that knockdown of PABPC1 expression significantly reduced miR-19a-3p levels in OCI/AML3-sEV and Blasts/mut-sEV, while miR-19a-3p levels in OCI/AML3 cells and Blasts/mut cells were almost unchanged, and the interaction between PABPC1 and miR-19a-3p in OCI/AML3 and Blasts/mut cells was further verified by miRNA pull-down and RIP assays, which indicated that PABPC1 plays a key role in miR-19a-3p encapsulation into sEVs.

Homeostatic regulation of protein synthesis plays a crucial role in hematopoietic stem cell differentiation and cellular transformation, and a major clinical challenge in the treatment of patients with HR-MDS is the progression to AML, which is driven by developmentally abnormal hematopoietic stem and progenitor cells with different metabolic changes (178). Trans-transport RNA-derived fragments (tRFs) are small emerging non-coding RNAs that are commonly altered in tumors (179). Studies have shown that stem cell-enriched pseudouridylation (Ψ) of tRF isoform 2 (mini-tRF containing 5’ terminal oligopurine (mTOG)) selectively inhibits abnormal protein synthesis programs, thereby promoting implantation and differentiation of hematopoietic stem and progenitor cells in patients with myelodysplastic syndromes. On the basis of mTOG-Ψ targeting PABPC1, the interaction of mTOG and PABPC1 impeded the recruitment of PAPI1 as revealed using isotope exchange proteomics. The translation of the transcript sharing pyrimidine-rich sequence (PES) and the 5’ terminal oligopyrimidine bundle (TOB) encoding the mechanical component of the protein was strongly inhibited in the 5’ untranslated region, while mTOG dysregulation resulted in increased aberrant translation of 5′ PES mRNA in malignant MDS-HSPC, which was clinically associated with leukemic transformation and reduced patient survival (68, 180, 181).

### PABPC1 in lung tumor

Lung cancer has the second highest cancer incidence and the second highest cancer-related mortality rate worldwide, with approximately 85% of lung cancer cases being non-small cell lung cancer, and non-small cell lung cancer consisting primarily of lung adenocarcinoma and squamous cell carcinoma of the lung (21, 182). Although tyrosinase inhibitors and immunotherapy have shown significant survival benefits for some patients, their 5-year overall survival rate is less than 15%. Researchers have recently identified that the MKRN3-PABPC1 pathway plays an important role in lung cancer pathogenesis (183). Germline mutations in the makorin ring finger protein 3(MKRN3) gene cause central precocious puberty (CPP), which is epidemiologically associated with various diseases in adulthood, including cancer (31–33) (184). Li et al. analyzed public data from cancer genomics studies and found recurrent inactivation of genomic MKRN3 aberrations in non-small cell lung cancer (183). low levels of MKRN3 expression were associated with poor patient survival, and in both *in vivo* and ex vivo, MKRN3 reduced cell growth and proliferation. Further proteomic screening by mass spectrometry identified PABPC1 as the major substrate of MKRN3. the tumor suppressive function of MKRN3 is dependent on its E3 ubiquitin ligase activity, and MKRN3 missense mutations were found to impair MKRN3-mediated PABPC1 ubiquitination in patients. the A203/F206/P208 of MKRN3 is a key interaction residue mediating PABPC1 ubiquitination, and the second RRM in PABPC1 is required for PABPC1 ubiquitination. Non-protein hydrolytic ubiquitination attenuates its binding to mRNA3’ poly(a), thereby inhibiting total protein synthesis and maintaining lung cancer cells with limited proliferative capacity. MKRN3 restoration induces cell cycle arrest in the G2/M phase, an effect also due to less PABPC1 binding to the poly(a) tail of CCNB1 mRNA, downregulating its protein levels. binding, downregulating its protein levels. This study contributes to the understanding of the genetic drivers of PABPC1 ubiquitination in non-small cell lung cancer.

### PABPC1 in other cancers

In addition to the above mentioned tumors, PABPC1 has clinical and prognostic relevance to other tumors. For example, Eisermann et al. reported that PABPC1 is a novel AR co-regulator that regulates AR function and subcellular localization in prostate cancer cells (185). knockdown of PABPC1 inhibited the proliferation of AR-positive prostate cancer cells. An et al. demonstrated that PABPC1 was upregulated in gastric cancer and its high expression was significantly associated with poorer overall and disease-free survival (25). et al. further demonstrated that PABPC1 knockdown induced apoptosis in gastric cancer cells through upregulation of pro-apoptotic and downregulation of anti-apoptotic proteins, and that miR-34c was a target of PABPC1. PABPC1 acts as an oncogene promoting the growth and invasion of ovarian cancer cells in ovarian cancer partly through regulation of the EMT process (23).

Histone acetylation modification is one of the major types of histone modifications for chromatin structural remodeling and transcriptional regulation. Acetylation neutralizes the positive charge of lysine and unfolds chromatin structure, thereby attenuating DNA-histone interactions and enhancing transcriptional activity. Dong et al. found that SNHG14 regulates the expression of PABPC1 through H3K27ac8, which activates the Nrf2 signaling pathway and promotes breast carcinogenesis and resistance to trastuzumab (24). et al. demonstrated by microarray that H3K27ac was enriched in the PABPC1 promoter region of hepatocellular carcinoma cells and that SNHG14 regulated PABPC1 expression in hepatocellular carcinoma cells through H3K27ac. The study showed that PABPC1 interacted with BDNF-AS and increased its expression by stabilizing the expression of BDNF-AS, and overexpression of both inhibited proliferation, migration and invasion of glioblastoma and promoted apoptosis (30). PABPC1 interacts with lncRNA-PAGBC in gallbladder cancer (186), enhancing the stability of the latter, while lncRNA-PAGBC competitively binds miR-133b and miR-511 and activates the AKT/mTOR pathway to promote tumor growth and metastasis.

Further study of the relationship between PABPC1 and other tumors is important for understanding the mechanisms by which PABPC1 promotes tumor development and its potential role in tumor therapy (**Table 2**).

**Table 2:** The expression and role of PABPC1 in multiple tumors.

Cancer type	Aberrant expression	Role	Associated clinical feature	Biological function	Target
Ovarian cancer	Up	Oncogenic	OS	viability, invasion and migration, EMT	
Gastric cancer	Up	Oncogenic	OS, DFS, Depth of invasion, Lymph node metastasis, pTNM, Vessel invasion	viability	miR-34c
Esophageal squamous cell carcinoma	Up	Oncogenic	lymph node metastasis, pathological stage, tumor recurrence, outcome, OS	proliferation, apoptosis, invasion, migration, angiogenesis	eIF4G, IFN/IFI27, miR-21-5p, CXCL10
Hepatocellular cancer	Up	Oncogenic	Tumor number, OS, AFP, TNM stage	proliferation, angiogenesis	AGO2, miR-183, miR-124, SNHG14, PTEN/PI3K/Akt, PTEN/VEGF
Prostate cancer	Up	Oncogenic	Recurrence	proliferation	AR
Glioblastoma	Down	Tumor suppressor		proliferation, migration, invasion, apoptosis	lncRNA-BDNF-AS/RAX2/DLG5, Hippo
Endometrial cancer				proliferation	IGF2BP1, PEG10
Breast cancer				trastuzumab resistance, proliferation, apoptosis, invasion, migration	lncRNA-SNHG14, Nfr2, HO-1
Non–small cell lung cancer				cell cycle, apoptosis, proliferation	MKRN3, eIF4G, CCNB1

## Conclusions

The central dogma of molecular biology has guided scientists’ research for a long time, and the targets of tumor research have focused on the final protein function. As scientific research continues, scientists have discovered that the translation process from mRNA to protein also plays an important role in tumor progression and drug resistance. Changes in mRNA translation in tumors are more extensive than those in transcription downstream of aberrant signaling pathways, and in response to oncogenic signaling or microenvironmental stressors, alterations in intracellular mRNA translation allow rapid changes in the proteome, increasing cancer cell adaptation, leading to tumor formation, spread metastasis and treatment resistance (15).There is no doubt that PABPC1 plays an important role in mRNA translation and stability as an RNA binding protein. Over the past two decades, The issue surrounding the importance of PABPC1 and poly(A) in translation has been controversial. Through exhaustive experiments in different cell types and cell conditions, researchers have demonstrated that poly(A) tail length and translation efficiency are coupled by PABPC1 at early stages of embryonic development, while at later stages, this relationship shifts to promote mRNA stability (53).

Most past studies on PABPC1 in tumors have focused on detecting gene copy number and/or protein expression of PABPC1 in different types of tumor tissues or cells, and its high expression in some solid tumors is associated with poorer patient prognosis (23–30, 142, 161, 183, 185–190). PABPC1 selectively regulates transcripts by interacting with non-coding RNAs such as long-stranded non-coding RNAs, microRNAs specific translation and expression of specific oncogenic proteomes, enhancing cancer cell plasticity and thus promoting therapeutic evasion of cancer progression (24, 28–30, 167, 186).

Studies of PABPC1 initially revealed its broad function as a tumor and metastasis-promoting protein, and PABPC1 gene overexpression was associated with abnormalities in tumor cell proliferation, apoptosis, invasion and distant metastasis. The effect of PABPC1 on cell proliferation and migration was verified by knocking down and overexpressing PABPC1 in ovarian cancer cell lines, and the deletion of PABPC1 significantly inhibited the viability and invasiveness of SKOV3 cells, while the upregulation of PABPC1 in A2780 cells showed the opposite result (23). Similarly, PABPC1 depletion in gastric cancer cells BGC823, MKN-45 and MGC803 reduced cell proliferation rate and colony-forming activity, and tumor xenografting assays suggested that PABPC1 knockdown significantly inhibited gastric cancer growth *in vivo (* 191). Similar studies include metastatic duodenal cancer (192), glioblastoma (30), and hepatocellular carcinoma (27–29). All these data provide evidence supporting the possible involvement of PABPC1 in tumorigenesis as an oncogene.

These current findings are far from sufficient, and more features and biological functions of PABPC1 protein remain to be explored. Further studies are urgently needed to demonstrate the molecular and cellular mechanisms by which PABPC1 plays a role in the development of different tumor cells, and to elaborate the tumor-promoting effects of PABPC1, which may pave the way for the development of inhibitors or agonists and the treatment of tumor patients with abnormal expression of PABPC1.

## Data availability statement

The original contributions presented in the study are included in the article/supplementary materials. Further inquiries can be directed to the corresponding author.

## Author contributions

YQ and QJ wrote the manuscript, surveyed and analyzed the literature. MW contributed with intellectual expertise and supervised this work. All authors contributed to the article and approved the submitted version.

## Funding

The work was supported by the Outstanding Scientific Fund of Shengjing Hospital (Grant No. 201705). And this work was also supported by the 2019 Liaoning Provincial Education Department Project (ZF2019017).

## Acknowledgments

We thank Mr. Chou for his thoughtful suggestions.

## Conflict of interest

The authors declare that the research was conducted in the absence of any commercial or financial relationships that could be construed as a potential conflict of interest.

## Publisher’s note

All claims expressed in this article are solely those of the authors and do not necessarily represent those of their affiliated organizations, or those of the publisher, the editors and the reviewers. Any product that may be evaluated in this article, or claim that may be made by its manufacturer, is not guaranteed or endorsed by the publisher.
